# The Educational Differentiation of African Birth Timing

**DOI:** 10.1111/sifp.12281

**Published:** 2025-02-03

**Authors:** Margaret Frye, Sara Lopus

## Abstract

As educational access has expanded across Africa, birth timing has remained quite stable. Using data representing 51 birth years and 34 countries, we show that these modest aggregate changes mask more dramatic changes within educational groups. Over time, educational attainment has become an increasingly salient predictor of birth timing, as highly educated women have delayed first births and lengthened subsequent birth intervals more. The educational differentiation of birth timing also varies across contextual factors (educational access and family planning effort). In recent cohorts, women of all educational levels have experienced earlier first births in higher education contexts, suggesting that entry into motherhood is influenced by relative position within one's peer group. Aggregating across educational levels, however, women experience later first births in higher education contexts, driven by the greater share of highly educated women. For women at all levels of educational attainment, first birth timing is responsive to family planning context; in aggregate, women in countries with high family planning investments become mothers one year later than those in countries with lower family planning efforts. Notably, highly educated women lengthen their second birth intervals more when education and family planning are widely available, suggesting further potential for public investments to enable women to achieve their reproductive preferences.

## BACKGROUND

Beginning in the second half of the twentieth century, much of the Global South experienced a fertility transition, in which women began to have fewer children and delay their entry into motherhood (Bongaarts 2017, Bongaarts and Hodgson 2022; Lima et al. 2018; Spoorenberg 2010). In Africa, however, the onset of the fertility transition came later and has proceeded slower than expected in numerous countries (Bongaarts 2017; Shapiro et al. 2013), first births continue to occur at younger ages than elsewhere (Bongaarts, Mensch, and Blanc 2017), and women have not adopted the parity‐specific birth spacing of other world regions (Moultrie, Sayi, and Timaeus 2012).

Africa's fertility trajectory has diverged from other world regions despite substantial improvements in educational access and family planning investments, both of which are understood to be key contextual‐level drivers of fertility transition (Kravdal 2002; Bongaarts and Hodgson 2022; Lutz and Samir 2011). Around the world, educational outcomes and family formation behaviors are closely intertwined (Bongaarts, Mensch, and Blanc 2017), and educational attainment is almost universally associated with completed family size (Lutz and Samir 2011). However, predictions that the spread of mass education would bring about widespread changes in fertility patterns (Caldwell 1980) have not come to pass in Africa, where fertility outcomes at a given level of educational expansion remain higher than in other less‐developed countries (Bongaarts 2017). Additionally, although higher use of contraception is associated with lower levels of fertility across the Global South (Liu and Raftery 2020), aggregate fertility rates in Africa have changed less than might be expected considering the widespread investments in family planning on the continent (Bongaarts 2017).

Africa's relatively modest aggregate changes in fertility behavior may mask more dramatic changes among some groups of women if fertility outcomes have diverged over time. Some subgroups of women may be delaying their first births and waiting longer to have another child than they previously did, while other subgroups may be having earlier first births and shorter birth intervals. Indeed, a growing body of knowledge in family demography focuses not on population‐level trends but on changing variability in reproductive behavior, exploring how this variability relates to other types of social stratification within populations (Castro Torres, Batyra, and Myrskylä 2022; Frye and Lopus 2018; Batyra 2020; Stoebenau et al. 2021). In South America, for example, social class differences and educational disparities in fertility timing have widened over time (Castro Torres 2021; Batyra 2020). Castro‐Torres et al. (2022) find that in Global South settings characterized by economic inequality, a bifurcation has occurred: some women are waiting much longer to have a child even as other women continue to transition to motherhood early. We know less about this topic in sub‐Saharan Africa, though recent work has found the age at first birth has become more variable in West Africa over time, as measured by an interquartile range (Batyra and Kohler 2022). And, across African countries that have experienced rising economic inequality in recent decades, elite versus nonelite women have experienced “diverging destinies” as they adopt increasingly distinct fertility behaviors (Stoebenau et al. 2021).

Building upon this recent work focusing on changing variability across subgroups rather than population‐level trends, we examine whether the *educational* differentiation of fertility timing in sub‐Saharan Africa has changed over time and across contexts. Looking across five decades and 34 African countries, we examine whether the timing of first and second births has become more differentiated by educational attainment in recent cohorts, or, conversely, whether women with different educational attainment have grown more similar in their birth timing. Next, we harness cross‐national variation in educational access and family planning investments to examine how women with different levels of education respond to variation in these contextual measures when timing their first and second births.

### The Timing of Births

Studies of birth timing generally investigate starting (the age at which a person becomes a parent), spacing and postponement (the length of intervals between subsequent births), and/or stopping and curtailment (the age at which one's last child is born). Each of these measures can be impacted by social conditions including cultural norms, economic and educational opportunities, and the availability and acceptability of contracepting. First birth timing, for example, is influenced by age at first marriage (Bongaarts et al. 2017), which itself can be shaped by the prevalence of schooling (Frye and Lopus 2018) and beliefs about the incompatibility between marriage and one's studies (Frye 2017; Lloyd and Mensch 2008).

Predictors of birth intervals are particularly varied and complex, due in part to the contribution of both spacing and postponing behaviors. When mothers engage in “spacing,” they aim to delay pregnancy until a certain length of time has passed since the most recent birth. Mothers who engage in “postponing,” on the other hand, aim to delay pregnancy due to reasons unrelated to their fertility histories (Johnson‐Hanks 2007; Moultrie, Sayi, and Timaeus 2012; Timæus and Moultrie 2020). We foresee various pathways through which educational attainment could influence both spacing and postponement. Highly educated mothers might be motivated to space their births due to the belief that they will be better able to invest in children who are born in slower succession; these same highly educated mothers may postpone childbearing due to higher opportunity costs associated with having a child if they are focusing in their careers (Goldin and Katz 2002), delaying until they have achieved a particular standard of living, or facing a great deal of uncertainty amid changing socioeconomic conditions (Johnson‐Hanks 2004). Alternatively, less‐educated mothers may pursue postponement in response to poverty or union instability. Due to our focus on the timing of first and second births, we are not able to disentangle these two processes, which would require comparing birth intervals across parities; for this reason, we use the more general term of “lengthening” birth intervals throughout the paper. Our results are also unlikely to be influenced by widespread stopping or curtailment, which would be more pronounced for higher parity births and in lower fertility populations.

### The Role of Educational Context

Education has expanded dramatically across Africa during the past half‐century, with educational access for girls increasing in virtually all African countries (Frye and Lopus 2018; Lopus and Frye 2018). Alongside these changes in educational prevalence, concurrent changes in marriage and family formation processes are theorized to be driven by two types of pathways. First, education impacts demographic events through individual pathways, such that the experience of going to school causes a person to adopt distinct behaviors. For example, completing secondary school may lead women to delay marriage. Second, education impacts demographic behaviors through societal or contextual pathways, such that people's behaviors are influenced by the availability of schooling in their community. For example, widespread prevalence of secondary education might bring about changes in women's labor force participation and status, which may impact behaviors regardless of a woman's own level of educational attainment.

Importantly, a third pathway from education to demographic behavior is also possible, in which individual educational attainment and societal educational context interact, such that a given level of educational attainment is associated with one set of behaviors in one context but with a different set of behaviors in another (Esteve and Florez‐Paredes 2018; Frye and Lopus 2018). In other words, variation in the extent of a country's educational expansion can influence the social meaning of a given educational credential and, in turn, the extent to which a woman's demographic behaviors are shaped by her educational attainment.

Given the well‐documented links between individual‐level educational attainment and fertility behaviors (e.g., Bongaarts, Mensch, and Blan 2017; Lutz and Samir 2011), we expect women who completed secondary school to have later first births and longer birth intervals than their peers who attended little or no school. Although this pattern of educational differentiation is likely to exist regardless of context, we expect the *extent* of educational differentiation to vary contextually. Specifically, looking across the range of educational contexts, from those in which very few girls completed primary school to those in which virtually everyone achieved that educational milestone, we foresee four possibilities:

1.1 Highly educated women delay first births and/or lengthen their subsequent birth intervals more in high‐education contexts.
1.2 Highly educated women delay births and/or lengthen birth intervals more in low‐education contexts.
1.3 Women with little or no formal schooling delay births and/or lengthen birth intervals more in high‐education contexts.
1.4 Women with little or no formal schooling delay births and/or lengthen birth intervals more in low‐education contexts.

The first and third scenarios would be observed if a given educational level shapes behaviors principally through its association with social, cultural, and economic conditions that influence childbearing. We might expect, for example, that highly educated women would be most motivated or able to delay their entry to motherhood in contexts in which female labor force participation is high, women are permitted to make their own decisions, and it is culturally permissible for young women to remain unmarried and/or childless while finishing school or building a career. All of these conditions are likely to coincide with contexts in which education is widespread (Scenario 1.1). Women with no formal schooling might also be most likely to delay their births in these high‐education scenarios if the cultural conditions associated with widespread schooling trickle down even to those who never attended any school in a “bandwagon effect” (Scenario 1.3). Such findings would align with those observed for marital timing across sub‐Saharan Africa (Frye and Lopus 2018).

The second and fourth scenarios above would be observed if a given educational level shapes behaviors principally through its capacity to indicate relative status and mark women as more advantaged or disadvantaged than their agemates. When a woman is highly educated in a context in which education is rare, for example (Scenario 1.2), we may expect her to adopt particularly unique behaviors (i.e., delayed births) not because of the effect of the education per se but because she is such an unusual person—a member of a small elite. Along these same lines, when a woman has little education in a context in which school participation is very low (Scenario 1.4), we may not expect her to give birth at a particularly early age because low educational attainment education does not act as a salient marker of relative disadvantage when it represents the modal status. Such findings would align with those in Latin America, where Esteve and Florez‐Paredes (2018) determined that the timing of childbearing remained constant over time within strata of relative education, indicating that it was a woman's relative educational position, and not her years of educational attainment, that shaped her fertility behaviors.

### The Role of Family Planning Context

Family planning programs are theorized to influence contraception use through two principal pathways (Easterlin and Crimmins 1985; Cleland et al. 1994). First, in contexts where fertility regulation is not yet widespread, there can exist a “latent demand” for family planning, in which women not currently using contraception would do so if it were more accessible and less costly. Family planning investments can lower constraints on contraceptive behavior, whether they be social (e.g., stigma), psychological (e.g., stress), or practical (e.g., distance or time), leading to an uptick in contraception use (Cleland et al. 1994; Easterlin and Crimmins 1985; Entwisle et al. 1984). Through this pathway, family planning programs can meet preexisting demand for contraceptives. Second, increased family planning effort can alter norms, economies, and the status of women, causing women to reduce or delay their reproductive aspirations (Bongaarts 2011, 2020; Easterlin and Crimmins 1985). Through this pathway, expanded family planning programs can create demand for contraceptives among women who otherwise would not have desired to limit, space, or postpone their births.

As above, regardless of family planning context, we expect women who completed secondary school to have later first births and longer birth intervals than women who attended less school, in part due to the widespread positive association between educational attainment and contraceptive use (Ainsworth, Beegle, and Nyamete 1996; Bongaarts 2010; Kirk and Pillet 1998). More educated women are likely to know more about family planning (Cochrane 1979) and to find contraception more acceptable (Cleland and Wilson 1987) than their less‐educated peers. However, we expect the magnitude of this difference to vary across family planning environments.

Our hypothesized associations between educational attainment and family planning focus on convergence and divergence of birth timing across educational groups rather than on increasing/decreasing delays within any one group. This is because, unlike in this case of educational context mentioned above, we do not anticipate any scenarios in which a group of women would have earlier births or shorter birth intervals in contexts with more access to family planning.^1^ Instead, we expect all relationships between birth timing and family planning effort will be either neutral or positive, which means we foresee just three possibilities instead of four. Looking across the range of family planning contexts, those three possibilities are as follows:

2.1 The birth timing of highly educated and less‐educated women *diverges* with increases in family planning.
2.2 The birth timing of highly educated and less‐educated women *converges* with increases in family planning.
2.3 The size of the difference between the birth timing of highly educated and less‐educated women *remains stable* with increases in family planning.

The scenario of divergence (Scenario 2.1) would be observed if highly educated women are more receptive to increased family planning effort than women with less schooling. This could be due to higher levels of latent demand among highly educated women: to the extent that macro‐level contraception accessibility facilitates micro‐level fertility preferences (Entwisle et al. 1984), we would expect increased family planning effort to bring about a steeper delay in birth timing for women who are highly motivated to limit, space, or postpone their births (presumably the most highly educated women). It is also possible that the expansion of family planning programs creates the disproportionate demand for contraception among highly educated women, as they might be most responsive to shifting gender norms and the expansion of economic opportunities for women (Goldin and Katz 2002). Moreover, having gone to school could promote media literacy, thereby facilitating educated women's access to curricular materials designed to alter fertility preferences as part of the nation's family planning effort (Mutumba 2022; Westoff and Bankole 1997).

The scenario of convergence (Scenario 2.2) would be observed if less‐educated women are more receptive to increased family planning effort than highly educated women. One reason for this finding could be if family planning investments provide information that reduces barriers to contracepting. In this case, in settings with very low family planning effort, contraception may be accessible only to women who had been to school, but in settings with higher family planning effort, all women would have the information necessary to access contraception (Entwisle et al. 1984). Put another way, as contraceptive prevalence expands, fertility regulation would transition from an “innovative behavior” of the educational elite to a “habitual” behavior practiced by women of all educational backgrounds, in accordance with the social diffusion hypothesis (Martin 1995). Whereas highly educated women may delay births regardless of the cultural context and social acceptability of contraception, less‐educated women may have latent demand that would be responsive to government‐sponsored communications heralding the benefits of smaller family sizes.

Finally, if women are equally receptive to increased family planning effort regardless of their educational attainment, the size of the gap in birth timing between highly educated and less‐educated women would remain stable across family planning contexts (Scenario 2.3). This could indicate that educational attainment does not influence a woman's receptiveness to family planning programs or the barriers she faces in using contraception. Alternatively, the finding of stable difference across contexts could mean that various factors are operating simultaneously (e.g., creating new demand among highly educated women and reducing barriers among less educated women), resulting in a net effect of no difference due to equal increases in contraceptive uptake by educational group as family planning efforts increase.

## METHOD

### Data

Since the 1980s, the Demographic and Health Surveys (DHS) program has conducted nationally representative household‐based surveys with a focus on population and reproductive health (ICF 1990–2021).^2^ DHS questionnaires and survey implementation protocols are standardized, allowing for the comparison of outcomes across countries and over time. We pool all available DHS surveys from 34 sub‐Saharan African countries to create a dataset of 754,222 women aged 30–49 at the time of the survey (Table 1). Most DHS surveys sample women aged 15–49; however, for this project, we restrict this age range to 30–49 to allow most women to complete their education and have their first birth before being included in the analysis. Although a hazard model of first birth timing does not require all women to have completed their first birth, restricting the dataset to a minimum age at which nearly all women who will have a first child have already done so allows us to also examine the second birth interval without biasing results by excluding women who will ultimately go on to have a first birth. Surveys were conducted between 1990 and 2021, with women's birth years ranging from 1940 to 1991. Because the timing of surveys varies by country, the span of birth years for which data are available also varies by country (Table 1).

**TABLE 1:** Descriptive statistics

Country	*N*	Observed birth years	Percentage of women who completed primary school or higher		Family planning effort	
			Earliest cohort	Latest cohort	1972	2014
West Africa						
Benin	40,889	1946–1988	5.1%	18.1%	10	43.7'
Burkina Faso	18,266	1943–1980	2.5%	13.0%	0	48.9'
Chad	12,987	1947–1985	0.3%	16.9%	0	46.3
Cote D'Ivoire	8579	1944–1982	5.5%	23.8%	0	45.0
Gambia	17,632	1963–1990	15.2%	48.1%	9.7'	54.4'
Ghana	13,605	1943–1984	28.0%	65.9%	10	53.4
Guinea	20,362	1950–1988	7.8%	18.2%	0	47.7'
Liberia	14,818	1957–1990	10.4%	50.7%	10	45.8
Mali	36,108	1946–1988	3.9%	17.9%	0	51.5
Niger	14,768	1942–1982	0.8%	5.1%	0	43.7'
Nigeria	77,149	1940–1988	11.9%	58.3%	7	41.0
Senegal	67,607	1943–1989	8.5%	21.4%	0	54.1
Sierra Leone	24,308	1958–1994	13.8%	28.6%	0	46.8'
Togo	8229	1948–1984	7.9%	34.4%	0	51.4
East Africa						
Burundi	10,682	1960–1987	8.2%	28.2%	10.5'	55.8
Comoros	3261	1946–1982	1.7%	47.1%	‐	‐
Ethiopia	31,663	1942–1986	2.0%	21.4%	0	59.2
Kenya	26,506	1943–1989	14.7%	66.9%	20	59.4'
Madagascar	32,948	1942–1991	13.0%	41.4%	0	47.2
Malawi	30,692	1942–1986	3.7%	39.6%	0	46.9
Mozambique	14,592	1947–1981	1.3%	20.2%	0	43.7
Rwanda	36,677	1942–1990	2.1%	38.3%	0	73.7
Tanzania	24,352	1941–1986	5.2%	69.3%	10	46.2
Uganda	28,050	1945–1986	11.3%	51.3%	0	49.8'
Central and Southern Africa						
Angola	5444	1965–1986	25.6%	49.0%	0	41.7'
Cameroon	24,430	1941–1988	10.8%	60.1%	0	38.8
Congo	7659	1955–1982	51.9%	76.3%	0	38.0
Congo DRC	11,704	1957–1984	27.5%	48.8%	0'	39.8
Gabon	6104	1950–1982	41.4%	78.6%	4.9'	49.6'
Lesotho	8579	1955–1984	44.5%	76.7%	0	42.0
Namibia	13,270	1942–1983	26.5%	81.6%	2.1'	53.5
South Africa	19,316	1948–1986	52.0%	95.4%	12.3'	60.8
Zambia	26,870	1946–1988	14.3%	60.2%	0	44.2
Zimbabwe	16,116	1944–1985	27.5%	88.7%	10	59.7

NOTE: ‘ marks countries for whom missing family planning effort (FPE) in 1972 and/or 2014 was interpolated based upon the country's nonmissing FPE scores between 1982 and 2009. See the Online Appendix for more information on FPE scores, including the interpolation methods. No FPE data are available for Comoros.

To complement the DHS data, we also use country‐level measurements from the family planning effort (FPE) index.^3^ The FPE has been collected every five to ten years between 1972 and 2014 in most countries of interest and is standardized to allow for comparisons over time and across countries (Kuang and Brodsky 2016; Ross and Smith 2010; Ross and Stover 2001). Unlike contextual measures of family planning output (e.g., contraceptive prevalence), which are endogenous to fertility preferences and behavior, the FPE focuses on family planning input. It quantifies a country's level of family planning effort as represented by the sum of its family planning policies, services, evaluation, and access according to a surveyed panel of key informants from within each country. Despite its appeal, this measure has important limitations. Most notably, the fact that it relies on the subjective evaluations of key informants, who differ across countries and change over time, limits both the precision of the estimates and the potential for researchers to corroborate this measure. Additionally, the measure is available only for a limited number of points in time for each country.

### Measures

Maternal age at first birth is calculated using the difference between the birth date of the first‐born child and the birth date of the mother, truncated to single years. We removed 522 women whose age at first birth was 10 or younger (0.06 percent of the sample); because of the low likelihood of having a live birth at this young age, we assume these cases more likely reflect inaccurate reporting or data entry errors. Second birth intervals were calculated using the difference between the birth date of the first‐born child and the birth date of the second‐born child, measured in months. The sample at risk of a second birth includes all primiparous women, removing 38,150 women who did not have a first birth.

To assess the role of individual‐level educational outcomes on birth timing, we categorize women's educational attainment using a credential‐based categorical measure: *no formal education* (for women who did not attend school), *some primary* (attended but did not complete primary school), *completed primary* (completed primary school and may have attended some secondary school), and *completed secondary or higher* (completed secondary school and may have attended or completed postsecondary school). We favor this categorical measure because it recognizes the important role of achieving educational milestones in shaping social status and livelihood opportunities. As an additional benefit of our chosen categorical measure, the focus on credentials allows us to meaningfully standardize educational outcomes across countries that have differing years‐to‐credential requirements (e.g., six years to complete primary school in Rwanda and Ghana, compared with seven years in Uganda and eight years in Kenya).

Other methods of measuring educational attainment would capture different dimensions of educational stratification; however, due to data limitations, heterogeneity in educational contexts within our dataset, and conceptual considerations related to how education can be expected to relate to fertility timing, we consider these alternatives to be less desirable for our purposes here. For example, using a continuous measure of the highest year of school completed would allow us to parse out the effects of finer gradations of educational attainment, but this method would treat each additional year of school as if it had a similar impact on fertility behaviors, whether that year yielded a diploma or not and whether it is typically completed during early childhood or late adolescence. A measure of total years spent in school, especially coupled with the age at which a woman left school, would allow us to capture differences in the duration of exposure or the proportion of a woman's youth spent in school. However, the DHS does not include this measure, and due to very high rates of repetition and delayed entry, the highest grade attained is not a reliable indicator of years spent in school in sub‐Saharan Africa (Ikeda and García 2014; Crouch et al. 2022). Alternatively, a relative measure of education (showing where in the distribution a person's schooling attainment falls in the distribution of her peers) would allow us to capture changes over time in how the educational “elite” structure their childbearing regardless of how long a woman needs to stay in school to achieve more education than most of her peers (see Stoebenau et al. 2021). However, when looking across 50 years and 34 countries, as we do here, an analysis of relative educational position would be hindered by the presence of numerous cohorts in which the vast majority of the population has never been to school. Further, this approach would prevent us from examining the relationship between individual educational attainment and contextual‐level educational prevalence.

To measure educational context, we calculate the percentage of women aged 25 or older within each country‐cohort who completed at least primary school. We construct two five‐year birth cohorts for each decade (e.g., 1980–1984 and 1985–1989) and pool all women born in the 1940s into one 10‐year cohort because of sample size limitations for these earliest cohorts. When calculating the percentage of women in each cohort who completed primary education, we use DHS survey weights to allow these estimates to be nationally representative. Compared with measuring the percentage who completed secondary school, as is sometimes done in higher education contexts, the percentage who completed at least primary school better reflects variations in educational access in the African context, which includes cohorts at very early stages of educational expansion. On the other hand, compared with measuring the percent who attended any school at all (e.g., Frye and Lopus 2018), we favor completion of at least primary school as the contextual measure for this research question because of the potential trade‐offs that adolescent girls may face between entering motherhood and staying in school during adolescence (Liu and Raftery 2020).

We use the FPE index as our measure of country‐level family planning context. To align FPE (a period measure) with our cohort‐based analyses, we assign each woman her country's FPE measurement from the time closest to the year in which she was 20 years old to reflect conditions around the time that many women enter marriage and begin motherhood. We exclude Comoros from all models incorporating FPE scores because there are no FPE data available for that country. For the other countries that have any missing FPE values between 1972 and 2014, we calculate the difference between the country's available FPE scores and the mean of all other countries’ FPE scores during those same periods; missing values were then imputed by adding the country‐specific difference to the period mean. Compared with other imputation approaches (e.g., country‐specific means or linear interpolations), this method generates imputed values that follow the general trajectory of FPE scores observed over time in nearly all African countries: low FPE scores in the 1970s and early 1980s, an uptick in family planning effort in the 1980s, and relative stability from the 1990s onward (Online Appendix Figure A1).

### Analytic Strategy

To begin, we model the age at first birth over time (birth year) using flexible parametric survival models, which make use of cubic splines to smooth the baseline log cumulative hazard function and are typically more accurate than the Cox proportional hazards approach (Royston and Parmar 2002; Rutherford, Crowther, and Lambert 2015). To account for time‐invariant differences in country‐level contexts, this model includes country fixed effects. For timing of second births, we replicate this approach, once again using flexible parametric hazard spline regression models to predict the length of the interval between women's first and second births with country fixed effects. The second‐birth models are limited to women who experienced a first birth (i.e., primiparous women) and include an additional control for age at first birth. To assess the role of individual‐level educational attainment and how this association changes over time, we next replicate each of these models (predicting age at first birth and the length of the second birth interval) including an additional control for educational attainment categories and an interaction between educational attainment and birth year.

Next, we model birth timing (age at first birth and the length of the second birth interval) across educational context, measured using the proportion of women in each country‐cohort who completed at least primary school. Rather than controlling for time‐invariant country‐level differences using fixed effects, these models include our two contextual‐level variables of interest: the FPE index and the percentage of women within each country‐cohort who completed primary school or higher. We first run these models for the entire population, without differentiating by educational attainment categories, to generate estimates for entire birth cohorts of women living in a particular educational context. To allow for the effects of educational context to vary across time, we interact educational context with birth year. Next, to examine educational differentiation across educational contexts, we run an additional model for each birth timing outcome, including the same controls as the undifferentiated educational context models and additionally controlling for educational attainment categories and interacting educational attainment with birth year. To allow for heterogeneity in the effect of educational context by educational attainment categories, we also interact the educational context measure with individual‐level educational attainment. Finally, we model birth timing across family planning context, following the same approach that we used for educational context, but interacting FPE index (rather than educational prevalence) with birth year and individual‐level educational attainment category.

For all first‐birth models, we display our results as the “midpoint” age (in years) by which 50 percent of all women had given birth to a first child, calculated from survival curves (Batyra and Kohler 2022). For second births, we display the midpoint interval (in months) by which 50 percent of all primiparous women had given birth to a second child, calculated from survival curves. Although similar to the concept of a median, this midpoint approach also includes data points for women who have not yet—and perhaps never will—experienced the birth in question (i.e., women who “survive” within the hazard model framework). When calculating the midpoint ages at first birth and midpoint birth intervals, we assign all control variables not displayed in the graph to their median values. For the models that include country fixed effects, we approximate a median country effect by plotting values for the country whose fixed effect coefficient is closest to the median of all country fixed effects in the model. In the Online Appendix, we explain our use of country and survey weights to adjust for variation in sampling sizes relative to population sizes across countries and over time. There, we also present the regression results for all models (Online Appendix Table A1) and the results of a robustness check in which we remove those country‐cohorts with imputed FPE values (Online Appendix Table A2).

## RESULTS

### The Expansion of Education and Family Planning Across Africa

**FIGURE 1:**
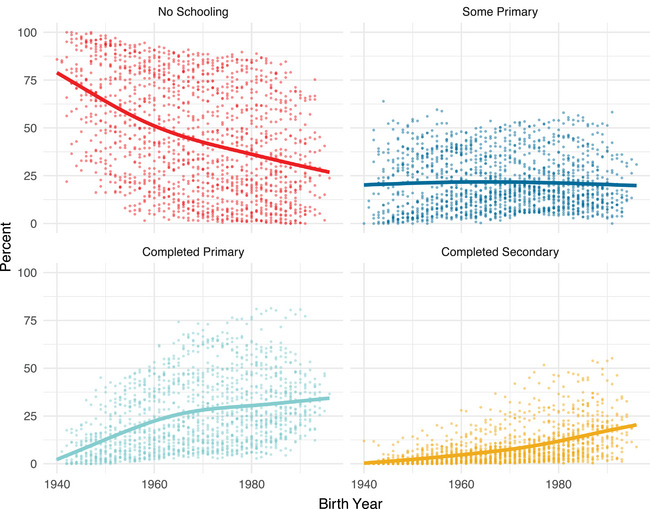
Educational composition by birth year across 34 African countries NOTE: These educational composition measures are constructed using all women aged 25 or higher and thus include some data points not represented in our birth timing models (which are restricted to age 30 or above). All estimates of educational composition were constructed using DHS survey weights.

**FIGURE 2:**
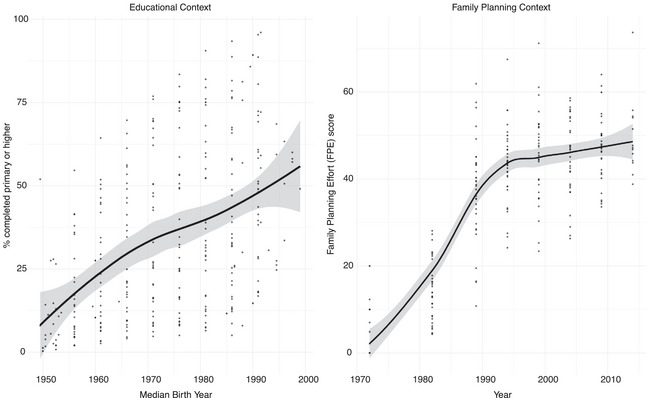
Educational and family planning context across 34 African countries over time NOTE: The educational context measure is constructed using all women aged 25 or higher and thus includes some data points not represented in our birth timing models (which are restricted to age 30 or above). Educational context measure was constructed using DHS survey weights. Likewise, the family planning effort graph includes all available data points for each country, but some of the more recent data points are excluded from our models due to our age restriction of 30 or above.

Educational access grew in all sub‐Saharan African countries over time (Table 1) while also demonstrating enormous heterogeneity across the continent within all birth cohorts (Figures 1 and 2). Over time, it has become increasingly common for women to complete primary or secondary school and increasingly uncommon for women to have attended no school at all (Figure 1). Educational access in West Africa lags behind the rest of the continent; Central and Southern African countries tend to have the highest levels of educational access (Table 1).

As educational access has grown over time in all sub‐Saharan African countries, so too has family planning effort, with steep increases between the 1970s and 1990s and more modest increases in recent decades (Table 1 and Figure 2). Across Africa, the mean FPE index increased from around 5 points in 1972, when women from the 1950s birth cohort were entering motherhood, to around 50 points in 2014, when women born in the 1990s were entering motherhood (Figure 2). At any given point in time, FPE scores have been quite variable across the continent (Table 1 and Figure 2).

### Educational Differentiation in Birth Timing Over Time

**FIGURE 3:**
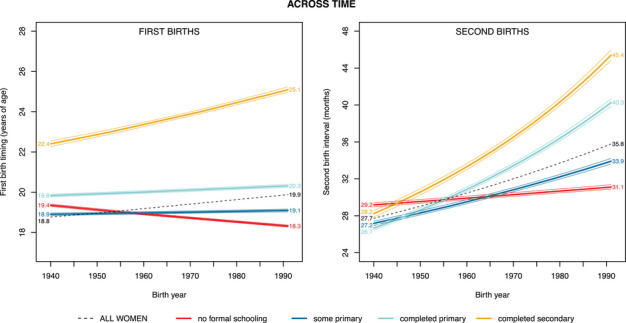
Midpoint age at which 50 percent of all women had experienced a first birth (left) and midpoint interval at which 50 percent of primiparous women had experienced a second birth (right), by birth year and educational attainment, across 34 African countries (weighted estimates) NOTE: Here, we display trends for the country closest to the median value of country‐level fixed effects, which was Burkina Faso for both first births and second births. For the second birth model, age at first birth is held at the median value for all women. Faint lines represent 95 percent confidence intervals.

Aggregating across sub‐Saharan Africa and across all levels of educational attainment, first births became marginally later over time: the predicted age at which half of all women had given birth (i.e., the midpoint age at first birth) grew by roughly one year over the span of 51 years, from 18.8 years of age for women born in the 1940 birth cohort to 19.9 years of age in the 1991 birth cohort (Figure 3, left panel, dashed black line). Disaggregating these trends by educational attainment reveals that women's educational attainment became an increasingly distinguishing factor in the timing of first births. Comparing secondary‐educated women born in 1940 to those with no formal education, the difference in the midpoint age at first birth in each educational category had given birth was 3.0 years; for women born in 1991, this difference had grown to 6.8 years (Figure 3, left panel). In fact, women with no formal education have begun to have children at *earlier* ages over time, with the midpoint age at first birth falling by around a year over the study period. In contrast, women who attended school have increasingly delayed their first births as time has progressed. The birth timing of secondary school graduates has most steeply diverged from the other educational groups, with the midpoint age at first birth increasing by nearly three years over the study period.

Birth intervals have also lengthened over time, with the predicted interval at which half of all primiparous women had given birth to a second child (i.e., the midpoint second birth interval) increasing by eight months over the course of the study period, from 27.7 months in the 1940 birth cohort to 35.8 in the 1991 birth cohort (Figure 3, right panel, dashed black line). Consistent with our findings for first birth timing, women's educational attainment became an increasingly distinguishing factor in the timing of second births (Figure 3, right panel). Comparing secondary‐educated women born in 1940 to those with no formal education, the difference in the midpoint second birth interval was only 1.6 months; by 1991, this difference increased to 14.3 months. Women in all educational groups delayed the birth of their second child more over time, but the magnitude of the delay was proportionate to women's level of educational attainment, ranging from an increased interval length of two months for women with no schooling to an increase of 17 months for women who completed secondary school. Like the secondary school graduates, women who had completed primary school also experienced dramatically longer birth intervals over time.

Although education played an increasingly prominent role over time in shaping the timing of both first and second births, the nature of educational divergence differed across parity. In particular, the baseline conditions among women born in the 1940s were quite different for first and second births. In these earliest birth cohorts, secondary‐educated women experienced their first births at dramatically later ages than their less‐educated peers (Figure 3, left panel). In comparison, in these same early birth cohorts, the length of the second birth interval was almost indistinguishable across educational groups (Figure 3, right panel). In other words, although the most highly educated women always delayed their entries into motherhood, once they were mothers, early cohorts of women tended to time their subsequent births very similarly to everyone else. Today, that is no longer the case: both first birth timing and second birth intervals are dramatically delayed for women with more education. We also note that the birth timing trajectory for women who completed their primary, but not secondary, educations is most similar to that of less educated women for first births and most similar to that of more educated women for second births. Having completed primary school therefore appears to have emerged over time as a salient predictor of second‐birth timing.

### Differentiation in Birth Timing Across Educational Context

In the results above, we document the increasingly important role of educational attainment over time in determining women's birth timing across all of sub‐Saharan Africa. Next, we move from investigating patterns across time, holding national context constant, to investigating educational differentiation in birth timing across the spectrum of educational contexts, from country‐cohorts in which few women had completed primary school to country‐cohorts in which most had done so. Although our models include data from all observed birth years, we plot the midpoint estimates for the birth year value “1990” to depict the most recent interplay between birth timing and context.

**FIGURE 4:**
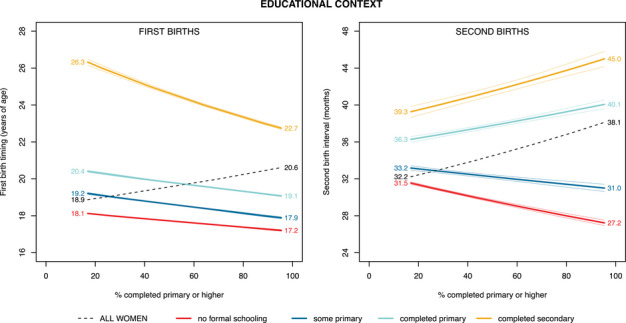
Midpoint age at which 50 percent of all women born around 1990 had experienced a first birth (left) and midpoint interval at which 50 percent of primiparous women had experienced a second birth (right) by educational context and educational attainment, across 33 African countries (weighted estimates) NOTE: Outcomes are plotted across the range of educational contexts experienced by women born between 1985 and 1996. Birth year is held at 1990; FPE score is held at the median value observed for women born between 1985 and 1996; for the second birth model, age at first birth is held at the median value for women in this same birth cohort. Faint lines represent 95 percent confidence intervals.

Aggregating across all levels of education, the overall timing of entry into motherhood among women born around 1990 was moderately later (1.7 years) in the highest education contexts compared to contexts where educational access was at its lowest levels (Figure 4, left panel, dashed black line). Disaggregating by educational attainment, higher educational attainment was consistently associated with delayed entries into motherhood, and educational context played a dramatic role in shaping first birth timing within a given educational group. However, for first births, we do not see much evidence of a *differential* response to educational expansion by educational groups; comparing across contexts, women in all educational groups experienced earlier births in contexts with more educational access than in contexts where education was less widespread, as indicated by the downward sloping lines for each educational group (Figure 4, left panel, Scenarios 1.2 and 1.4). We do note some modest differences in responsiveness to educational conditions across educational attainment categories, as reflected by the different slopes in the colored line**s**: for women with completed secondary education, women enter motherhood 3.6 years earlier in the highest education contexts, whereas for those women with no formal schooling, this difference is less than one year. The upward‐sloping line for “all women” amid downward‐sloping lines for each educational group can be explained by the changing educational composition of women, with a larger proportion in the higher education groups as we move from left to right along the *x*‐axis (Figure 1).

Turning next to second births, for women born around 1990, we find that greater educational access was once again associated with later overall birth timing, with a lengthening of around six months in the midpoint second birth interval in contexts with the highest levels of educational access in comparison with contexts where educational access was the least widespread (Figure 4, right panel, dashed black line). When disaggregating by educational attainment, however, dramatic differences emerge for the educational differentiation of second births in comparison to the patterns described for first births above. Highly educated women experienced later births in contexts in which education was widespread (Figure 4, right panel), consistent with Scenario 1.1. For the women with little or no formal education, however, higher educational settings were associated with shorter birth intervals (Scenario 1.4), as evidenced by the downward‐sloping lines.

### Differentiation in Birth Timing Across Family Planning Context

**FIGURE 5:**
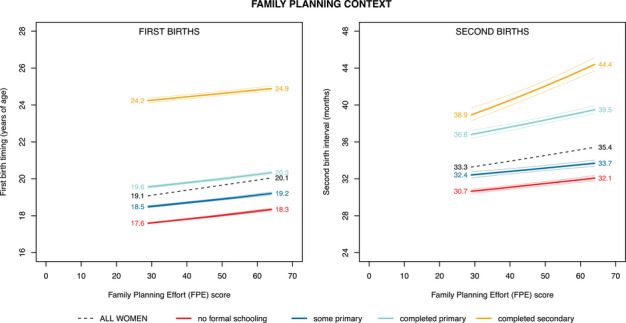
Midpoint age at which 50 percent of all women born around 1990 had experienced a first birth (left) and midpoint interval at which 50 percent of primiparous women had experienced a second birth (right) by family planning context and educational attainment, across 33 African countries (weighted estimates) NOTE: Outcomes are plotted across the range of family planning contexts assigned to women born between 1985 and 1996 (i.e., the contexts recorded in the year closest to that in which they were 20 years old). Birth year is held in 1990; educational context (percentage of women who completed primary school or higher) is held at the median value observed for women born between 1985 and 1996; for the second birth model, age at first birth is held at the median value for women in this same birth cohort. Faint lines represent 95 percent confidence intervals.

Turning next to family planning context, we see that the first birth timing of women born around 1990 was responsive to family planning effort. First births occur, in aggregate, one year later for women living in countries with the highest FPE scores than for those in the countries with the lowest observed FPE scores (Figure 5, left panel, dashed black line). Disaggregating by women's educational attainment, we find that within a given family planning context, higher educational attainment was associated with delayed entries to motherhood. However, the slopes of the lines for women in each educational group are roughly parallel, meaning that neither divergence nor convergence is observed as FPE increases (Scenario 2.3). Although the upward slope of all lines suggests that women delay their births more in contexts with more investments in family planning, the lack of convergence or divergence suggests that the extent of family‐planning‐related delay of first births is not associated with the women's level of educational attainment.

For second births, we see that women born around 1990 are only marginally responsive to family planning context in aggregate, with the second birth interval being around two months longer in the countries with the highest investments in family planning than in the lowest FPE contexts (Figure 5, right panel, dashed black line). As family planning investments increase, a pattern of educational divergence is observed (Scenario 2.1), indicating a modest educational difference in responsiveness to family planning context. Whereas women with little or no formal education lengthen their second birth intervals by only a month or two when family planning effort is higher (Figure 5, right panel), highly educated women lengthen their birth intervals more (by over five months) in high‐FPE contexts.

Comparing the dashed black lines in Figures 4 and 5, we see more variation in aggregated birth timing across educational context than across FPE. Focusing first on the left panels of these figures, aggregated timing of first births varies by 1.7 years across educational contexts, compared with only 1.0 years across family planning contexts. Likewise, comparing across the right panels of these figures, aggregated timing of second births varies by about six months across educational contexts, compared with only two months across family planning contexts.

## DISCUSSION

Building on a recent body of literature investigating the changing heterogeneity of demographic events across status groups, this article investigated educational differentiation of first and second birth timing. Looking across time and controlling for time‐invariant differences in national contexts, we found strong evidence of educational divergence for both first and second births: across sub‐Saharan Africa, women's educational attainment became an increasingly salient determinant of both when a woman becomes a mother and how quickly she has another child. These diverging trends may help to explain the documented stability of fertility timing in the aggregate (Bongaarts, Mensch, and Blanc 2017; Shapiro et al 2013), with first births moving only 1.1 years over a 50‐year span. As highly educated women have delayed their entry into motherhood and subsequent births, their less educated peers are accelerating their own transitions, and these two trends counteract one another when the whole population is analyzed. These findings of divergence are consistent with previous scholarship documenting the increasing salience of educational (Batyra 2020; Bongaarts, Mensch, and Blanc 2017) and economic (Castro Torres, Batyra, and Myrskylä 2022; Stoebenau et al. 2021) inequality in shaping birth timing within both Latin America and Africa.

Our analyses also revealed notable differences between the extent of educational differentiation in first and second birth timing. In the earliest observed birth cohorts, there were already meaningful differences in the timing of first births across educational groups, such that women from the 1940s birth cohorts who completed secondary school became mothers years later than did women with no formal education. Interestingly, such a gap between the least and most educated women did not exist in the 1940s birth cohorts with regard to the timing of second births: regardless of educational status, women gave birth to their second child about the same length of time after their first births. The lack of an educational gap for second birth suggests that in this early birth cohort, although highly educated women delayed the onset of entry into motherhood, once they were mothers, their behaviors conformed to those of their less‐educated peer group, and they no longer exhibited distinct patterns in the timing of their subsequent births.

Both educational and family planning access have expanded across the continent, but they have done so unevenly, such that some women born in 1990 had educational opportunities similar to some other women born in 1950, and some women born in 1990 lived in countries with only slightly greater investment in family planning than some other women born in 1950. Looking across educational access and family planning contexts for women born in this most recent time period, we found that, in general, both of these contextual variables had some association with aggregate fertility timing, as evidenced by the upward‐sloping black dashed lines in Figures 4 and 5. From a policy perspective, the differing slopes of these dashed lines indicate that aggregate timing of first births appears to be responsive to both educational and family planning investments, whereas the timing of second births appears to be more responsive to investments in education.

Returning to the four scenarios we outlined above through which differences in educational access may shape the fertility timing of less‐ and more‐educated women, we found that for women born around 1990, educational attainment predicted the age of entry into motherhood principally through its ability to indicate relative status. Secondary‐educated women adopted particularly unique birth timings in lower education contexts where they were part of a smaller elite group; in contexts where more of their agemates reached the same level of education or surpassed them and attended universities, secondary‐educated women entered motherhood at an earlier age (Scenario 1.2); this pattern is depicted by the steeply downward‐sloping yellow line on the left side of Figure 4. We also found that women with no formal schooling exhibited more distinct behaviors (in this case, earlier births) when more of their agemates had completed primary school—that is, contexts in which their lack of schooling placed them in a uniquely disadvantaged minority (Scenario 1.4; depicted by the downward‐sloping red lines in Figure 4). In other words, as lack of schooling became a more salient marker of disadvantage, only the most disadvantaged women remained in this category, while other women, who may always have delayed their births regardless of educational context, entered different educational groups.

It is therefore women's relative rather than absolute educational attainment that influences fertility behaviors. These findings from Africa are consistent with those described elsewhere by Esteve and Florez‐Paredes (2018), who document the role of relative position in what they call a “stability paradox,” with aggregated birth timing remaining stable despite educational expansion. In the African case, the salience of relative status may help explain why predictions of rapid fertility transition have not come to pass despite widespread educational expansion (Bongaarts 2017; Caldwell 1980). That said, the overall effect of relative educational position in sub‐Saharan Africa is not powerful enough to cause full‐fledged stability in birth timing across educational context. Instead, compositional changes, with an increasing proportion of women in the more highly educated categories and a declining proportion of women in the lowest categories (Figure 2), offset the effects of relative status, such that the overall trend is one of later first births when educational access is greater (dashed black line in Figure 4).

In our examination of differentiation in the second birth interval across educational access, secondary‐educated women lengthened their birth intervals more in contexts where education was more widespread (Scenario 1.1, right side of Figure 4). This pattern is consistent with women in higher education settings being able to take advantage of access to social and economic changes including better labor market opportunities and higher female labor force participation, broader support in the population for women's autonomy and role in household decision‐making, and an increasing acceptability of preferences for smaller family size (Gyimah 2009; Rindfuss, Brewster, and Kavee 1996; Yu and Xie 2015). Among women with no formal education who were born around 1990, however, we once again found confirmation that educational attainment shaped birth timing primarily through its indication of relative status. Consistent with our finding for first births for this group, the interval between first and second births was shorter in higher education settings, where these no‐schooling women constituted a uniquely disadvantaged minority (Scenario 1.4, right side of Figure 4).

Moving now to our results examining heterogeneity in educational differentiation across family planning contexts, we showed that for first births, family planning effort impacted women equally across levels of educational attainment, such that living in a country with higher FPE was associated with a later age at first birth, regardless of individual‐level education (Scenario 2.3, left side of Figure 5). For second births, however, the pattern was different: highly educated women lengthened their intervals more in contexts with more family planning resources and greater availability of contraception, but women with little or no formal education did not (Scenario 2.1, right side of Figure 5). The positive relationship between educational attainment, birth interval length, and FPE is consistent with the existence of new or latent demand for longer intervals among highly educated women, perhaps due to their receptiveness to evolving gender norms and expanded economic opportunities for women (Goldin and Katz 2002). Whether met or created, this demand for longer intervals does not appear to exist among women who did not attend or complete primary school, indicating that as contraceptive prevalence expands, using contraception to lengthen birth intervals is largely an “innovative” behavior of the educational elite (Martin 1995). Unlike their better educated peers, less‐educated women time their second births essentially the same way regardless of family planning context.

Despite these contributions, our analysis is limited by the fact that educational context and FPE are both measured at the national level, preventing us from drawing finer geographic distinctions within countries. Although this analysis is focused on presenting results aggregated across sub‐Saharan Africa, future research should examine differences across region, colonial regime, and cultural and political context. Finally, our analysis is focused on documenting the differentiation of birth timing by educational attainment, and not on isolating the causal processes through which women of different levels of education come to exhibit these different reproductive behaviors. It is possible that schooling may be capturing differences in other markers of status, such as urban versus rural residence, income, or health, rather than the effects of going to school per se. Future research should work to apply more of a causal approach, possibly by limiting the analysis to a smaller geographic area where these potential confounding factors could be more effectively parsed out. We also focus our comparisons on the midpoint estimates for each educational group; future research might follow a promising line of research using the interquartile range (Batyra and Kohler 2022) and quantile regression (Batyra 2024) to examine differences in the variability of birth timing across educational groups.

For those interested in the policy applications of this research, we find that both educational investments and family planning investments have the potential to influence birth timing in the aggregate, although they do so in different ways. Public investments in schooling operate by changing the composition of the population, such that more women will attain levels of education associated with delayed entry into motherhood and lengthened birth intervals. Family planning investments, on the other hand, change the within‐group behaviors, such that all women—regardless of their educational attainment—delay and space their births more when family planning services are more widely available. These differing impacts on group composition (schooling investments) and within‐group behavior (family planning investments) show particular potential to influence birth timing when the two policies are adopted in combination (see also Bongaarts and Hardee 2019). However, when educational opportunities expand, women who do not attend any school may be left behind in terms of their ability to achieve their reproductive goals, and they could face enduring barriers to contraception or reproductive health resources. The fact that the birth spacing behaviors of these same women change only slightly with greater investments in family planning suggests either that the preferences of women who do not attend school are less responsive to family planning messages or that they are not fully able to take advantage of family planning resources.

## CONCLUSION

Over time, educational attainment has become an increasingly salient predictor of the timing of births across Africa, as highly educated women delayed their first births and lengthened their subsequent birth intervals more than their less‐educated counterparts. By the 1990s birth cohorts, contextual factors were meaningful predictors of birth timing, both within educational groups and across the full population. Although expanded educational access was associated with earlier onset of motherhood among both highly educated women and those who have never been to school, higher education contexts were still settings for later birth timing in the aggregate due to the higher proportion of highly educated women in these contexts. These findings provide insight into the mechanisms underlying Africa's unique fertility trajectory in comparison to that of the rest of the world.

## Supporting information



Online Supplemental Material

## Data Availability

All data used are publicly available and can be accessed through the following websites: Demographic and Health Survey Data (http://www.dhsprogram.com), the Family Planning Effort Index Data (http://www.track20.org/pages/data_analysis/policy/FPE.php), and the United Nations Population Division population size estimates for use in building the country weights (https://population.un.org/wpp/downloads?folder=Standard%20Projections&group=Population). The models presented in this paper were run using the flexsurv package in R (Jackson 2016). Code files are available upon request, please email the corresponding author.

## Notes

1 This pattern might be seen if there were a strong pattern of reverse causality, in which family planning investments were made in the highest need settings and if family planning programs had weak or nonexistent effects on contracepting behavior. However, we view this scenario as unlikely.

2 Data are available at www.dhsprogram.com

3 Data are available at www.track20.org/pages/data_analysis/policy/FPE.php
