# Supplementary material for: The Educational Differentiation of African Birth Timing
Source: Stud Fam Plann. 2025 Feb 3;56(1):87–111. doi: 10.1111/sifp.12281 (PMC11956804; doi:10.1111/sifp.12281)
Supplement: Supplementary file 1 — Online Supplemental Material [file SIFP-56-87-s001.pdf]

Online Supplemental Material

Survey Weights

All analyses presented in this paper use the individual-level women's survey weights provided by the Demographic and Health Surveys to make the data nationally representative of women. We account for variation in sample size relative to population size across countries and over time by multiplying the survey weights by the inverse of the sampling rate (with the sampling rate defined as the number of women aged 25-49 who were surveyed divided by the estimated population size for persons aged 25-49, Lopus and Frye 2020). To estimate annual population sizes, we use the United Nations World Population Prospects data (United Nations Population Division 2017). All reported estimates of mean values or proportions based on the full sample use these population-adjusted weights.

Model Results

Results from the models presented in the manuscript are in Tables A1 through A6.

Imputed Values for Missing FPE Scores

Family Planning Effort (FPE) scores are available for all countries except Comoros. Between 1994 and 2004, the FPE questionnaire transitioned from a long form (containing over 100 items, which were recoded and reduced into 30 scores) to a short form (containing only summary about the 30 scores). In 1999, which is the only year for which both the long form and short form were used, long and short FPEs were strongly correlated within sub-Saharan African countries (R=0.620). Our model uses the 1999 short scores and includes an FPE-year interaction term to account for systematic changes in FPE measurements over time.

Nearly all countries display a pattern of low FPE in 1972 and 1982, increasing FPE between 1982 and 1989, and relatively steady FPE from 1994 onward (Appendix Figure A1). When imputing values for the missing FPE scores, we therefore determined it was not appropriate to use linear interpolation methods, which would not take this overall pattern into account. Instead, we calculate the difference between the country's available FPE scores and the mean of all other countries' FPE scores during those same periods; missing values were then imputed by adding the country-specific difference to the period mean. Figure A1 displays the country-specific FPE scores, with black points representing periods for which data was available and red points depicting the values that were imputed in cases of missingness.

As a robustness check, we run versions of the models in which we remove country-cohorts with missing FPE scores. The results of those models are presented as dashed lines in Figures A2 through A5 and are compared with the full-sample results from the manuscript, which are depicted with solid lines. Although some small differences do emerge (e.g. later first births among the highly educated women in the robust models than the full-sample models), the paper's overall findings (directions of slopes, differences across educational groups) remain the same. The most notable difference is for the second-birth timing of the most highly educated women; the restricted sample suggests that their birth spacing is more responsive to educational context than does our full-sample model. In both the first- and second-birth models, the full-sample model provides a more conservative estimate of the aggregate response to educational context than does the reduced-sample model, as evidenced by the more gradual slope of the solid black line in Figures A2 and A4. Given the similarities in results across models, we choose to present the full-sample models in the manuscript to avoid losing around 11% of datapoints (632,464 first-birth events instead of 716,072; 580,021 second-birth events instead of 655,321) from our analyses.

Figure A1. Countries' Family Planning Effort (FPE) values, 1972 to 2014.

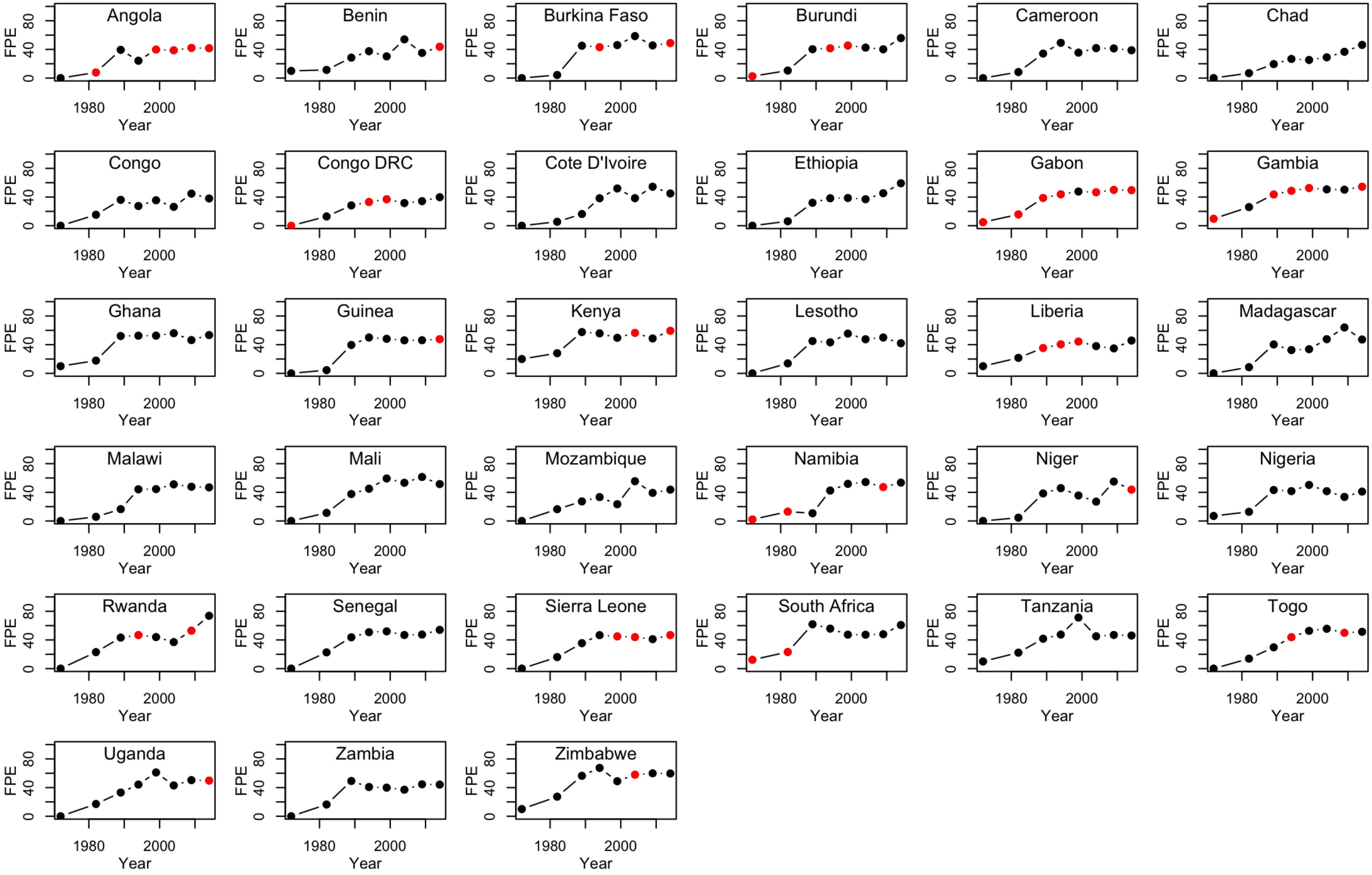

Notes: Black points represent those for which FPE data are available; red points represent imputed values for missing datapoints.

Figure A2. Robustness check: timing of first birth across educational contexts, 1990s cohort

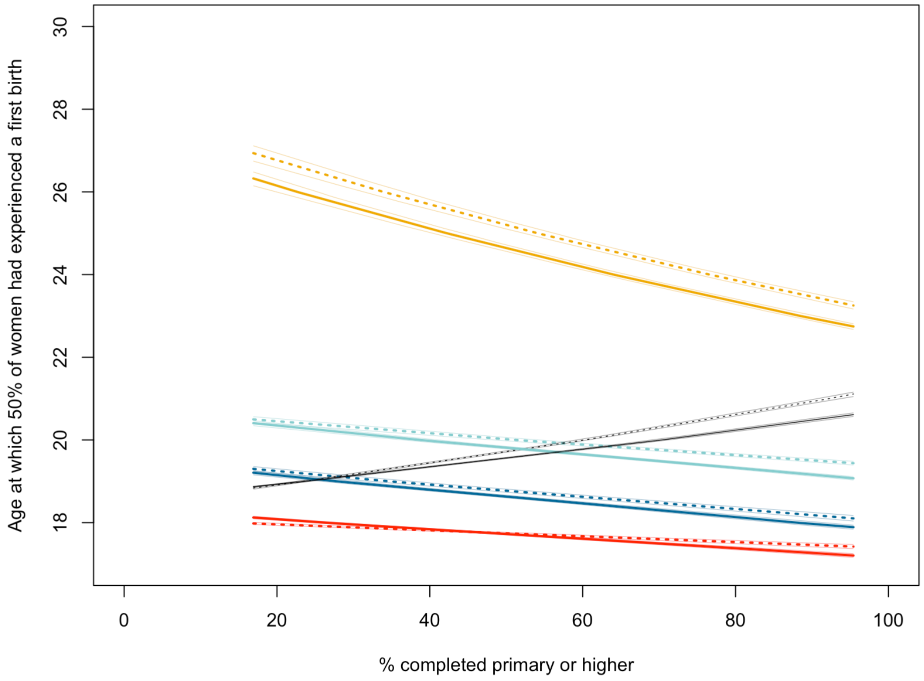

Notes: Solid lines represent the results of the full-sample model; dotted lines represent the results of a robustness check with a restricted sample in which country-cohorts with missing FPE scores were removed from analysis.

Figure A3: Robustness check: Timing of first birth across family planning contexts, 1990s cohort

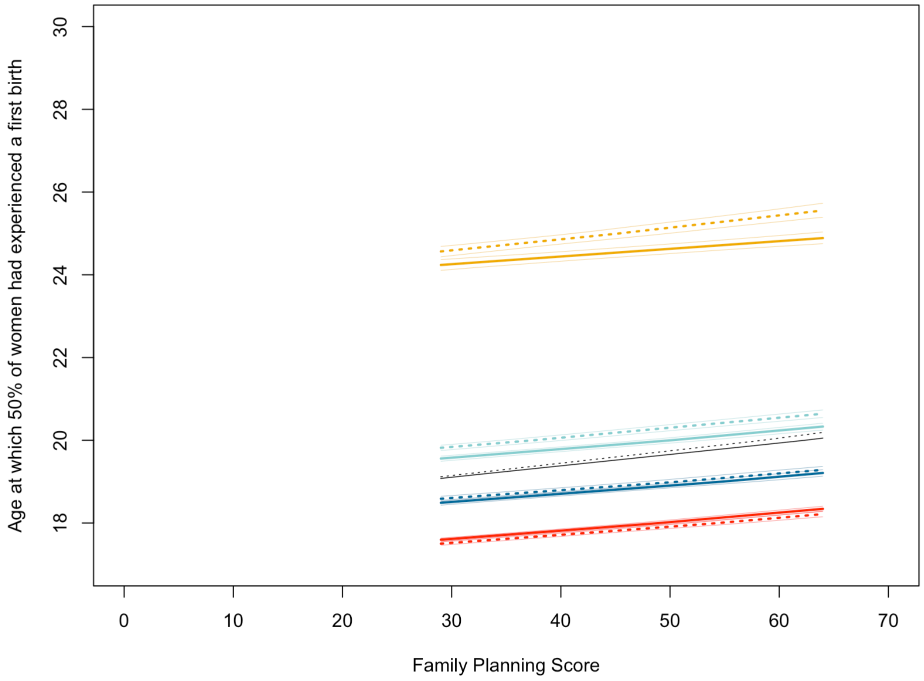

Notes: Solid lines represent the results of the full-sample model; dotted lines represent the results of a robustness check with a restricted sample in which country-cohorts with missing FPE scores were removed from analysis.

Figure A4. Robustness check: timing of second birth across educational contexts, 1990s cohort

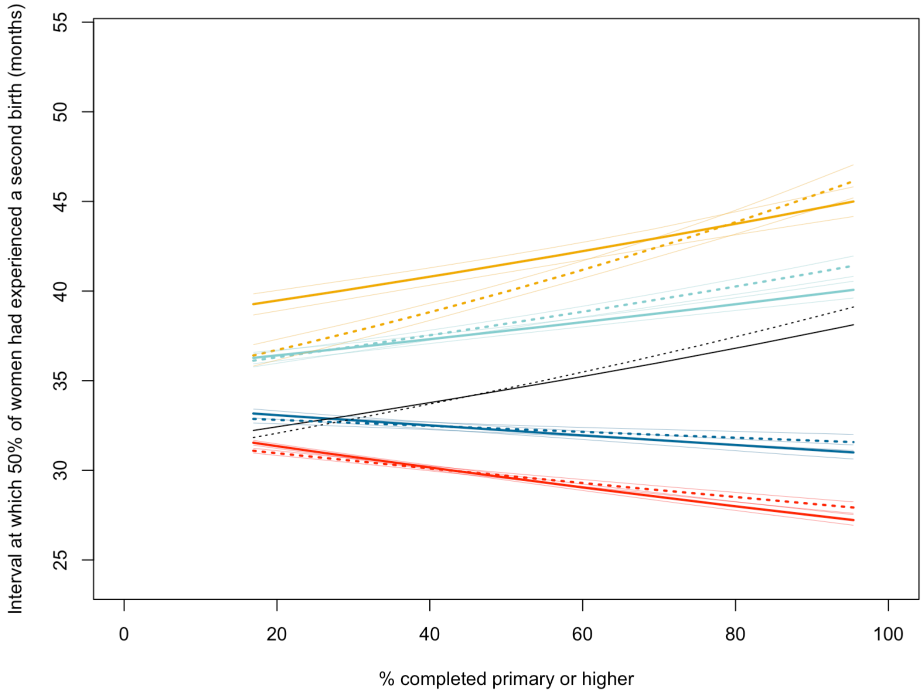

Notes: Solid lines represent the results of the full-sample model; dotted lines represent the results of a robustness check with a restricted sample in which country-cohorts with missing FPE scores were removed from analysis.

Figure A5: Robustness check: Timing of second birth across family planning contexts, 1990s cohort

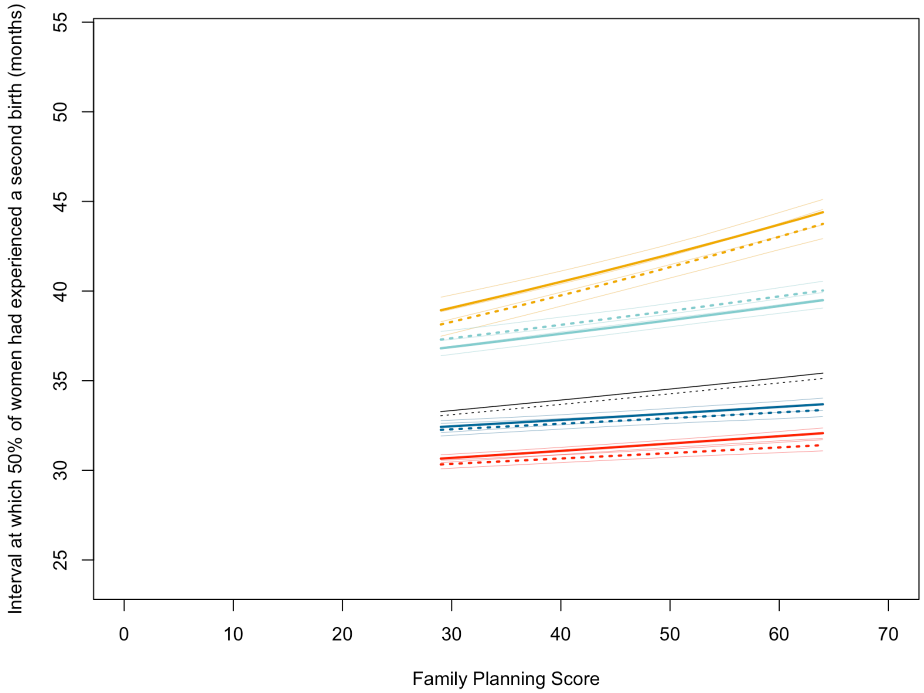

Notes: Solid lines represent the results of the full-sample model; dotted lines represent the results of a robustness check with a restricted sample in which country-cohorts with missing FPE scores were removed from analysis.

Table A1. First Births Across Time

	Aggregate Model			Educational Differentiation Model		
	Coef.	SE	p	Coef.	SE	p
Birth Year	-0.009	0.000	<0.001***	0.009	0.000	<0.001***
Ed. Attainment						
Some Primary				0.185	0.011	<0.001***
Completed Primary				-0.195	0.011	<0.001***
Completed Secondary				-1.038	0.017	<0.001***
Birth year * Ed. Attainment						
Birth year * Some Prim.				-0.011	0.000	<0.001***
Birth year * Comp. Prim.				-0.012	0.000	<0.001***
Birth year * Comp. Sec.				-0.022	0.000	<0.001***
N	754,222			754,222		
Events (first births)	716,072			716,072		

Table A2. First Births Across Educational Context

	Aggregate Model			Educational Differentiation Model		
	Coef.	SE	p	Coef.	SE	p
Birth Year	0.006	0.000	<0.001***	0.009	0.000	<0.001***
FPE	-0.005	0.000	<0.001***	-0.006	0.000	<0.001***
Ed. Context	-0.447	0.021	<0.001***	-0.415	0.027	<0.001***
Birth Year * Ed. Context	-0.007	0.001	<0.001***	0.020	0.001	<0.001***
Ed. Attainment						
Some Primary				0.184	0.012	<0.001***
Completed Primary				-0.086	0.013	<0.001***
Completed Secondary				-0.913	0.020	<0.001***
Birth year * Ed. Attainment						
Birth year * Some Prim.				-0.0138	0.000	<0.001***
Birth year * Comp. Prim.				-0.0173	0.000	<0.001***
Birth year * Comp. Sec.				-0.0320	0.001	<0.001***
Ed. Context * Ed. Attainment						
Ed. context*Some Prim				0.159	0.023	<0.001***
Ed. context*Comp Prim				0.056	0.021	0.007**
Ed. context*Comp Sec				0.402	0.026	<0.001***
N	750,961			750,961		
Events (first births)	713,177			713,177		

Table A3. First Births Across Family Planning Contexts.

	Aggregate Model			Educational Differentiation Model		
	Coef.	SE	p	Coef.	SE	p
Birth Year	0.010	0.000	<0.001***	0.012	0.000	<0.001***
FPE	0.002	0.000	<0.001***	-0.007	0.000	<0.001***
Educ. Context	-0.693	0.010	<0.001***	0.331	0.009	<0.001***
Birth Year * FPE	0.000	0.000	<0.001***	0.000	0.000	<0.001***
Ed. Attainment						
Some Primary				0.129	0.012	<0.001***
Completed Primary				-0.252	0.012	<0.001***
Completed Secondary				-1.06	0.018	<0.001***
Birth year * Ed. Attainment						
Birth year * Some Prim.				-0.012	0.001	<0.001***
Birth year * Comp. Prim.				-0.014	0.001	<0.001***
Birth year * Comp. Sec.				-0.028	0.001	<0.001***
FPE * Ed. attainment						
FPE * Some Prim.				0.001	0.000	0.001**
FPE * Comp. Prim.				0.002	0.000	<0.001***
FPE * Comp. Sec.				0.006	0.000	<0.001***
N	750,961			750,961		
Events (first births)	713,177			713,177		

Table A4. Second Births Across Time.

	Aggregate Model			Educational Differentiation Model		
	Coef.	SE	p	Coef.	SE	p
Birth Year	-0.016	0.000	<0.001***	-0.004	0.000	<0.001***
Age at First Birth	-0.032	0.000	<0.001***	-0.020	0.000	<0.001***
Ed. Attainment						
Some Primary				0.244	0.011	<0.001***
Completed Primary				0.312	0.011	<0.001***
Completed Secondary				0.112	0.020	<0.001***
Birth year * Ed. Attainment						
Birth year * Some Prim.				-0.010	0.000	<0.001***
Birth year * Comp. Prim.				-0.021	0.000	<0.001***
Birth year * Comp. Sec.				-0.023	0.001	<0.001***
N	716,072			716,072		
Events (second births)	655,321			655,321		

Table A5. Second Births Across Educational Context.

	Aggregate Model			Educational Differentiation Model		
	Coef.	SE	p	Coef.	SE	p
Birth Year	-0.008	0.000	<0.001***	-0.011	0.001	<0.001***
FPE	-0.002	0.000	<0.001***	-0.002	0.000	<0.001***
Ed. Context	-1.077	0.037	<0.001***	-0.832	0.045	<0.001***
Birth Year * Ed. Context	0.009	0.001	<0.001***	0.030	0.001	<0.001***
Age at First Birth	-0.035	0.001	<0.001***	-0.024	0.001	<0.001***
Ed. Attainment						
Some Primary				0.169	0.019	<0.001***
Completed Primary				0.379	0.022	<0.001***
Completed Secondary				0.123	0.038	0.002**
Birth year * Ed. Attainment						
Birth year * Some Prim.				-0.005	0.001	<0.001***
Birth year * Comp. Prim.				-0.013	0.001	<0.001***
Birth year * Comp. Sec.				-0.012	0.001	<0.001***
Ed. Context * Ed. Attainment						
Ed. context*Some Prim				-0.368	0.030	<0.001***
Ed. context*Comp Prim				-1.014	0.028	<0.001***
Ed. context*Comp Sec				-1.105	0.044	<0.001***
N	713,177			713,177		
Events (second births)	652,758			652,758		

Table A6. Second Births Across Family Planning Context.

	Aggregate Model			Educational Differentiation Model		
	Coef.	SE	p	Coef.	SE	p
Birth Year	-0.001	0.001	<0.01**	0.000	0.001	n.s.
FPE	0.001	0.000	n.s.	-0.001	0.000	0.034*
Ed. Context	-0.794	0.010	<0.001***	-0.321	0.012	<0.001***
Birth Year * FPE	0.000	0.000	<0.001***	0.000	0.000	<0.001***.
Age at First Birth	-0.035	0.001	<0.001***	-0.024	0.001	<0.001***
Ed. Attainment						
Some Primary				0.051	0.018	0.005**
Completed Primary				-0.063	0.020	0.002**
Completed Secondary				-0.352	0.035	<0.001***
Birth year * Ed. Attainment						
Birth year * Some Prim.				-0.005	0.001	<0.001***
Birth year * Comp. Prim.				-0.009	0.001	<0.001***
Birth year * Comp. Sec.				-0.004	0.001	0.002**
FPE * Ed. attainment						
FPE * Some Prim.				0.001	0.001	n.s.
FPE * Comp. Prim.				-0.001	0.000	<0.001***
FPE * Comp. Sec.				-0.005	0.001	<0.001***
N	713,177			713,177		
Events (second births)	652,758			652,758		

References

Lopus, Sara and Margaret Frye. (2020) "Intramarital status differences across Africa's educational expansion." *Journal of Marriage and Family* 82(2): 733-750.

United Nations Population Division. (2017) File POP/15-1: Annual total population (both sexes combined) by five-year age group, region, subregion, and country, 1950-2100 (thousands). *World Population Prospects 2017*. https://population.un.org/wpp/Download/Standard/Population/
